# Rapid Eye Movement Sleep Deprivation Produces Long-Term Detrimental Effects in Spatial Memory and Modifies the Cellular Composition of the Subgranular Zone

**DOI:** 10.3389/fncel.2016.00132

**Published:** 2016-05-30

**Authors:** Sofia Soto-Rodriguez, Gabriela Lopez-Armas, Sonia Luquin, Rodrigo Ramos-Zuñiga, Fernando Jauregui-Huerta, Oscar Gonzalez-Perez, Rocio E. Gonzalez-Castañeda

**Affiliations:** ^1^Laboratorio de Microscopía de Alta Resolución, Departamento de Neurociencias, Centro Universitario de Ciencias de la Salud, Universidad de GuadalajaraGuadalajara, México; ^2^Centro de Enseñanza Técnica IndustrialZapopan, Mexico; ^3^Laboratorio de Neurociencias, Facultad de Psicología, Universidad de ColimaColima, México; ^4^Departamento de Ciencias Básicas del Área de la Salud, División de Biotecnología y Salud, Tecnológico de Monterrey, Campus GuadalajaraZapopan, Mexico

**Keywords:** REM sleep deprivation, long term, spatial memory, proliferation, differentiation, apoptosis

## Abstract

Sleep deprivation (SD) affects spatial memory and proliferation in the dentate gyrus. It is unknown whether these deleterious effects persist in the long run. The aim of this study was to evaluate the proliferation, differentiation and maturation of neural progenitors as well as spatial memory 21 days after suffering SD. Sixty-day old male Balb/C mice were exposed to 72-h REM-SD. Spatial memory, cell fate, apoptosis and expression levels of insulin-like growth factor 1 receptor (IGF-1R) were evaluated in the hippocampus at 0, 14, and 21 days after SD or control conditions. After 21-days recovery period, memory performance was assessed with the Barnes maze, we found a significant memory impairment in SD mice vs. control (94.0 ± 10.2 s vs. 25.2 ± 4.5 s; *p* < 0.001). The number of BrdU+ cells was significantly decreased in the SD groups at day 14 (controls = 1.6 ± 0.1 vs. SD mice = 1.2 ± 0.1 cells/field; *p* = 0.001) and at day 21 (controls = 0.2 ± 0.03 vs. SD mice = 0.1 ± 0.02 cells/field; *p* < 0.001). A statistically significant decrease was observed in neuronal differentiation (1.4 ± 0.1 cells/field vs. 0.9 ± 0.1 cells/field, *p* = 0.003). Apoptosis was significantly increased at day 14 after SD (0.53 ± 0.06 TUNEL+ cells/field) compared to controls (0.19 ± 0.03 TUNEL+ cells/field *p* < 0.001) and at 21-days after SD (SD mice 0.53 ± 0.15 TUNEL+ cells/field; *p* = 0.035). At day 0, IGF-1R expression showed a statistically significant reduction in SD animals (64.6 ± 12.2 units) when compared to the control group (102.0 ± 9.8 units; *p* = 0.043). However, no statistically significant differences were found at days 14 and 21 after SD. In conclusion, a single exposition to SD for 72-h can induce deleterious effects that persist for at least 3 weeks. These changes are characterized by spatial memory impairment, reduction in the number of hippocampal BrdU+ cells and persistent apoptosis rate. In contrast, changes IGF-1R expression appears to be a transient event.

**Highlight** Sleep deprivation affects spatial memory and proliferation in the dentate gyrus. To date it is unknown whether these deleterious effects are persistent over a long period of time. We analyzed the effects of sleep deprivation in the hippocampus after 21 days of recovery sleep. Our findings indicate that after sleep recovery, the detrimental effects of SD can be observed for at least 2 weeks, as shown by a reduction in memory performance, changes in the hippocampal cellular composition and higher apoptotic rate over a long period of time.

## Introduction

Sleep is an essential physiological state in our daily life, which is a complex and highly organized neural process that involves several neurochemical systems synchronized with the environment. To date, there is not a consensus regarding the function of sleep, but some of its possible roles are energy restoration, thermoregulation, memory consolidation and cognitive homeostasis (Kandel et al., 2000; Cipolli et al., 2006).

Sleep deprivation is associated with weight loss, bacterial infections and, if SD persists for 2 or 3 weeks, the subject may die (Rechtschaffen et al., 1989). In the adult brain, SD of the REM phase produces important alterations in behavior and cognitive functions (Gonzalez-Castañeda, 2016). REM SD can produce aggressiveness (Martins et al., 2008), hyperactivity (Tufik et al., 2009) and memory deficits (Hagewoud et al., 2010). At the cellular level, REM SD produces changes in gene expression in cerebral cortex and other areas related to sleep regulation; these alterations include cellular stress, glial dysfunction (Guindalini et al., 2009) and, impairment in the memory consolidation and in the LTP (Cirelli, 2006) via NMDA receptors (Kopp et al., 2006).

The SGZ in the DG in the hippocampus is a neurogenic niche of the adult brain. Neurogenesis in the SGZ involves three main stages: proliferation, differentiation, and maturation (Christie and Cameron, 2006), which are regulated by several environmental and intrinsic factors, such as enriched environment or local growth factors. The neurogenic process is supported by neurotrophic factors such as BDNF, NGF and Insulin-like growth factor-1 (IGF-1). IGF-1 plays an important role in cell growth and development (Aberg et al., 2000). During early development, IGF receptors are highly expressed in neural stem cells and are essential for controlling neural proliferation, differentiation and maturation.

Previous studies have shown that 36-h REM SD significantly reduces the number of BrdU+ cells in the SGZ (Guzman-Marin et al., 2005, 2008; Mueller et al., 2008), an event that have been associated with memory dysfunction (Hagewoud et al., 2010; Havekes et al., 2012). These studies have been performed immediately after SD, but the long-term effects of SD in the neurogenic niche and hippocampal-dependent memory remain unknown. To address these questions, we evaluated the long-term effects of REM SD on the cell fate of neuronal precursors in the SGZ, the hippocampus-dependent memory, the SGZ apoptosis rate and the levels of IGF-1R. Our findings indicate that 72-h REM SD induces spatial memory impairment, reduction in the number of hippocampal BrdU+ cells and persistent apoptosis rate. Remarkably, these SD-related effects can be observed for at least 3 weeks after the SD exposure.

## Materials and Methods

### Animals and Housing Conditions

**FIGURE 1:**
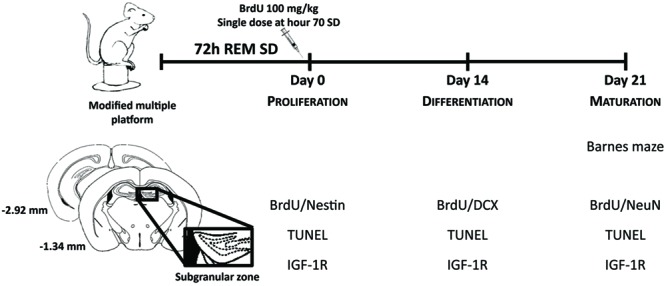
**Experimental design.** Mice were exposed to REM sleep deprivation for 72-h. Twenty-one days later, we evaluated spatial memory (Barnes maze). Cellular analysis was done at different time points to assess: proliferation, differentiation and maturation of SGZ neural progenitors, as well as apoptosis (TUNEL) and expression levels of IGF-1R.

The procedures described in the present study were approved by the Ethics Committee of the University of Guadalajara (CI-16610) in compliance with NIH regulations. Sixty-day-old Balb/C male mice were housed in polycarbonate cages (59.0 cm × 38.5 cm × 20.0 cm) and randomly divided into two groups: the sleep-deprived group and the control group. Before REM SD, animals were maintained in a 12 h light/12 h dark cycle (lights on at 08:00 A.M.), room temperature was set at 24°C ± 2 and humidity of 50% ± 20. Animals were then exposed to REM SD and control animals remained in the water environment housed in their own cages being allowed to sleep. To select the time point where the CORT levels showed less modification, we analyzed CORT serum levels at different time points of SD: 24-h, 48-h, and 72-h. Twenty-one days after 72-h SD, spatial memory task was analyzed in the Barnes maze. Two hours before finishing SD, we injected BrdU to label SGZ neural progenitors and sacrifice the animals at day 0, day 14, and day 21. Apoptosis and IGF-1R expression were analyzed at the same time points (**Figure 1**).

### Sleep Deprivation

Sleep deprivation of REM phase was done with the modified multiple platform paradigm (van Hulzen and Coenen, 1981). Briefly, 15 animals were placed in a large water tank containing 20 platforms. The water temperature was maintained at 26 ± 2°C throughout the study. Each platform was 2.5 cm wide, and 15 cm high. Tap water and food was available *ad libitum* in all platforms. To avoid overcrowding, the platforms outnumbered the animals; consequently, they could freely move around the platforms. The platforms were 2 cm above the water level; thus when animals reached the REM phase and lost their muscle tone, they fell into the water and forced to climb up on the platform. Previous experiments have demonstrated that the large platform used as standard control reduces approximately 80% of REM sleep (Machado et al., 2004). Control animals were housed in their cages allocated in the experimental room in order to be maintained in the same environment but privileging their normal sleep cycle. Animals were maintained in REM SD for up to 72-h, in a 12 h light/12 h dark cycle (lights on at 08:00 h).

### Spatial Memory Task (Barnes Maze)

After 72-h REM SD, the animals were returned to their cages and maintained under standard biotery conditions for 21 days. After this sleep recovery period, spatial memory was evaluated with the Barnes Maze (Barnes, 1979) (*n* = 6 mice per group). This maze consisted of a circular platform with 12 holes (5 cm diameter each) at the periphery; eleven of them were empty, but one of the holes had a dark shelter box underneath (**Figure 3A**). This maze helps measure the ability of the mouse to learn and remember the location of a target hole based on distal visual cues. This task relays on the individual decision of the mouse to escape from an aversive environment by using spatial (hippocampal-dependent) memory. All animals completed five assays per day (300 s each) for 3 days. When the animal did not find the target hole in 300 s, they were guided to the hole and let remained there for 60 s. We quantified the time spent to find the shelter hole (escape latency), time spent at each quadrant, and the number of visits and time spent at each hole. These visits were divided in short (less than 5 s) and long (5 or more seconds) stays. The escape latency was considered as parameter of spatial learning, whereas the time spent at each quadrant and the visits to each hole serve to analyze the strategies used by the animals to complete the task (Harrison et al., 2006).

### Corticosterone Analysis

To determine possible CORT fluctuations during the SD exposure, we quantified CORT concentration after 24-h, 48-h, and 72-h of REM SD. Animals were decapitated and blood was immediately collected into a sterile tube (*n* = 3 per SD time point). Blood serum was then obtained by centrifugation and analyzed with the ELISA kit *Enzyme Immunoassay for Corticosterone* (Oxford Biomedical Research) following the step-by-step protocol from the manufacturer. In order to avoid circadian variation, blood samples were obtained by decapitation always between 12:00 and 13:00 h.

### Bromodeoxyuridine Injections

BrdU is an analogous of thymidine that incorporates into DNA during cell division (Falconer and Galea, 2003). Two hours before the REM SD finished, we administered a single dose of 100 mg/kg i.p. BrdU (Sigma B5002), to label all progeny derived from the primary SGZ precursors. The labeled cells at this moment was used an indicator of proliferation after 72 h of SD. Thus, this single dose would allow us to follow up the lineage of cells through their development (proliferation on day 0, differentiation on day 14, and maturation on day 21). Animal sacrifices were made based on the maturation stages: immediately after SD, at day 14 and day 21 (**Figure 1**).

### Tissue Processing

Mice were sacrificed with a high dose of sodium pentobarbital (100 mg /kg body weight) before transcardial perfusion. For fluorescent microscopy (*n* = 5 animals per group), mice were perfused with 0.9% NaCl solution at 37°C followed by 4% paraformaldehyde in 0.1 M phosphate buffer, and the brains were post-fixed overnight at 4°C in the same fixative. Thirty five micrometer thick coronal sections were cut with a vibratome from -1.34 to -2.92 mm Bregma coordinates (George Paxinos, 2001). Slices for analysis were collected at every six slices and fluorescent immunostainings were performed as described below. For western blot, the groups were sacrificed at similar time points: immediately after REM SD, 14-days and 21-days post-SD (*n* = 4 per group). These groups were sacrificed by decapitation. Hippocampi were then dissected and frozen immediately at -80°C.

### Immunohistochemistry

DNA was denatured with 2N HCl during 10 min at 37°C followed by 10 min in boric acid 0.1 M (pH 8.5) at room temperature. The tissues were rinsed in PBS-Triton-X-100 0.03% three times for 5 min, and blocked with goat serum 10%. They were incubated with primary antibodies for 20 h at 4°C: rat anti–BrdU (1:400; AbD Serotec Cat# OBT0030 RRID: AB609568) plus mouse anti-Nestin (1:400; Millipore Cat# MAB353 RRID:AB94911), guinea pig anti-DCX (1:500, Millipore Cat# AB2253 RRID:AB1586992), or mouse anti-NeuN (1:400; Merck Cat# MAB377 RRID:AB11210778). After rinsing, the sections were incubated with appropriate secondary antibodies (1:1000, Alexa Fluor 488 for BrdU, Alexa Fluor 594 for cell markers; Invitrogen, Life technologies) for 60 min at room temperature. The brain tissues were mounted with fluorescence solution Vecta Mount (Vector, H-5000) and quantified under a fluorescence microscope Axio-observer D2 (Zeiss, Germany).

### Apoptosis Assay (TUNEL)

Brain tissues were permeabilized with the same treatment as the last section. A kit was used to label apoptotic cells *In Situ Cell Death Detection Kit, Fluorescein* (Roche Applied Science) using the TUNEL method, then mounted for observation under a fluorescence system.

### Western Blot

Four animals per group were decapitated and hippocampi were treated with protease tablets (complete mini EDTA-free, Roche, 04693159001) plus lysis buffer (Base Tris pH = 7.5, NaCl, NaF, Na_3_VO_4_, NP40) as described previously (Krishack et al., 2015; Shiwa et al., 2015; Galvez-Contreras et al., 2016). Briefly, the homogenate was centrifuged for 30 min at 14,000 rpm/4°C, and the supernatant was recovered and processed for Lowry protein assay (Lowry et al., 1951). Samples (30 μg total protein) were loaded in 7% SDS-polyacrylamide gels in SDS loading buffer (50 mM Tris-HCl pH 6.8, 2% SDS, 10% glycerol, 1% β-mercaptoethanol, 12.5 mM EDTA, and 0.02% bromophenol blue), electrophoresed and electrotransferred to immobilon-PSQ PVDF membrane (Merck-Millipore, ISEQ00010) in 39 mM glycine, 48 mM Tris-base, 1% SDS, 20% methanol, pH 8.3. After transfer, the membranes were blocked in 5% non-fat dry milk in TTBS (10 mM Tris–HCl, 150 mM NaCl, 0.1% Tween-20, pH = 7.5) for 2 h. Membranes were incubated overnight with primary antibodies against Anti-phospho-IGF-1R (1:200; Merck Cat# ABE332 RRID: AB11214503) diluted in TTBS. After washing three times with TTBS, membranes were incubated for 1 h at room temperature with 1:600 biotinylated anti-rabbit IgG (Vector laboratories Cat# BA-1000 RRID: AB2313606) HRP-conjugated IgG diluted in TTBS. Protein bands were visualized and quantified using Bio-Rad Quantity One detection system.

### Antibody Characterization

All antibodies used in the present research are listed in the **Table 1**. Anti-BrdU antibody recognizes chemical BrdU that is incorporated into DNA during synthesis phase of mitosis, replacing thymidine. Hence, BrdU expression exhibits nuclear localization (Kee et al., 2002). Anti-Nestin antibody recognizes intermediate filaments protein (class Type VI) of neuronal or dividing precursor nerve cells in the SGZ and is mostly expressed in nerve cells implicated in the radial growth of axons (Michalczyk and Ziman, 2005). Anti-DCX antibody recognizes a microtubule-associated protein expressed almost exclusively in immature neurons. Neuronal precursors express DCX shortly after exiting the cell cycle. DCX expression continues for 2–3 weeks until the neuroblasts turn into mature neurons (Brown et al., 2003). NeuN antibody (NeuN; clone A60) specifically recognizes the DNA-binding neuron-specific protein (NeuN), which is present in most CNS and PNS neuronal cell types of all vertebrates tested. NeuN protein distribution is apparently restricted to NeuN, perikarya and some proximal neuronal processes in both fetal and adult brain (Mullen et al., 1992).

**Table 1:** Antibody characterization for immunohistochemistry, ELISA and Western Blot.

Marker	Cell type	Antigen	Manufacturer, RRID	Concentration	Reference
BrdU	Dividing cells	Thymidine analog	AbD Serotec Cat# OBT0030 RRID:AB_609568	1:400	Seri et al., 2004
Nestin	Progenitor cells	Intermediate filaments	Millipore Cat# MAB353 RRID:AB_94911	1:400	Seri et al., 2004
Doublecortin	Immature neurons	Contributes to microtubule organization	Millipore Cat# AB2253 RRID:AB_1586992	1:500	Sevc et al., 2014
NeuN	Mature neurons	Nuclear epitope	Merck Cat# MAB377 RRID:AB_11210778	1:400	Seri et al., 2004
TUNEL	Apoptotic cells	Terminal deoxynucleotidyl transferase dUTP nick end	*In Situ* Cell Death Detection Kit, Fluorescein (Roche Applied Science # 11684795910)	As indicated by kit	Gavrieli et al., 1992
Anti-phospho-IGF-1R		Rabbit. Phosphorylated Tyr1161/Tyr1165/Tyr1166	Merck Cat# ABE332 RRID:AB_11214503	1:200	Saeed et al., 2007
Enzyme Immunoassay for Corticosterone		Rabbit. Anti-Corticosterone	Oxford Biomedical Research, EA 66	As indicated by kit	Arthaud et al., 2015

TUNEL detects DNA fragmentation that results from apoptotic signaling cascades. The assay relies on the presence of nicks in the DNA that can be identified by terminal deoxynucleotidyl transferase (TdT), an enzyme that will catalyze the addition of dUTPs that are secondarily labeled with a marker (Gavrieli et al., 1992). IGF-1 receptor (IGF-1R) is a disulfide-linked heterotetrameric transmembrane protein consisting of two alpha (130 kDa) and two beta (95 kDa) subunits. The IGF-1R is highly expressed in all cell types and tissues and is highly overexpressed in most malignant tissues where it functions as an anti-apoptotic agent by mediating signaling pathways that enhance cell survival (LeRoith et al., 1995). The IGF-1R activates alternative pathways for protection from apoptosis both during normal development and during stress or disease. CORT is a glucocorticoid secreted by the cortex of the adrenal gland. This hormone is produced in response to stimulation of the adrenal cortex by ACTH. CORT is a major indicator of stress and is the major stress steroid produced in non-human mammals. In addition to stress levels, CORT is believed to play a decisive role in sleep–wake patterns (Arthaud et al., 2015).

### Quantification

To quantify the number of double labeled cells located in the DG we assessed seven 35-μm sections, 210 μm apart (*n* = 5 animals per group) from -1.34 mm to -2.92 mm Bregma coordinates (George Paxinos, 2001). We counted new neurons in the SGZ of the DG, which is a one-cell-thick layer. The whole SGZ was quantified in all of the collected slices, ranging from one field in anterior slices (-1.34 mm coordinates from Bregma) to three fields in posterior slices (-2.92 mm coordinates from Bregma) to accomplish 30 microscopic field per animal. Double labeling was confirmed and quantified by matching cellular morphologies with clearly discernible nuclei and by analyzing non-overlapping high-power (400x) microscope fields (field area = 0.64 mm^2^). For imaging, a Zeiss Axio-Observer D1 microscope (Göttingen, Germany) and Axio-Vision 4.8.1 acquisition Software (AxioVision, RRID:SciRes_000111 Göttingen, Germany) was used. For every section, the percentage of co-localization of BrdU+ cells that co-expressed Nestin, DCX, or NeuN divided by the total number of BrdU+ cells per section multiplied by 100.

### Statistical Analysis

Data are expressed as mean ± standard error. For the statistical test we did a kurtosis analysis and found that our data displayed a non-parametric distribution. Therefore, for multiple comparison, we used the Kruskall–Wallis test and, for comparison between pairs, the Mann–Whitney “*U.*” In all cases, the *p* ≤ 0.05 value was chosen to establish statistically significant differences. The sample sizes used in this study were validated by calculating the effect size for each experiment and the respective statistical power (Thalheimer and Cook, 2002).

## Results

### CORT Concentrations Fluctuate during REM SD Exposure

**FIGURE 2:**
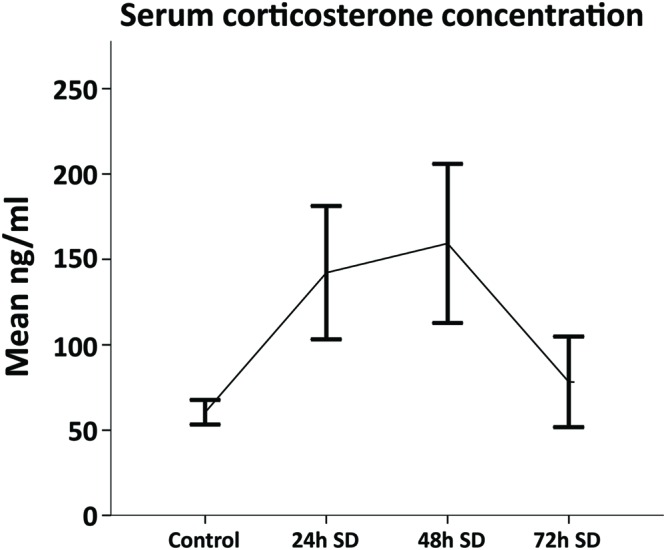
**Corticosterone serum levels throughout REM SD time course.** The levels of corticosterone concentration (mean ± SE) were increased by 24-h and 48-h as compared to controls (*n* = 3 per group). Although no significant differences were found amongst groups (Mann–Whitney “*U*”), the 72-h SD group showed corticosterone levels similar to the control group.

Corticosterone is one of the main stress biomarkers in animals (Dedovic et al., 2009). Since SD might be a stressful situation, we analyzed the concentration of serum CORT in animals exposed to REM SD (*n* = 3 per group). Animals were sacrificed by decapitation immediately after REM SD (24-h, 48-h, and 72-h SD), blood samples were immediately collected and CORT serum levels were analyzed by the ELISA method. We found that different periods of SD showed dissimilar CORT serum levels: 24-h SD (142.2 ± 33.8 ng/ml), 48-h SD (159.3 ± 40.31 ng/ml), and 72-h SD (78.25 ± 18.77 ng/ml) compared to non-SD-deprived controls (60.5 ± 6.30 ng/ml) (**Figure 2**). According to these findings, the maximum CORT serum levels occurred 48-h after SD, whereas the CORT concentrations decreased at 72-h SD. Therefore, to avoid as much as possible the effect of CORT levels on cell fate assessments, we decided to perform the cellular analysis with the group of 72-h REM SD.

### Spatial Memory Is Affected at Long-Term by SD

**FIGURE 3:**
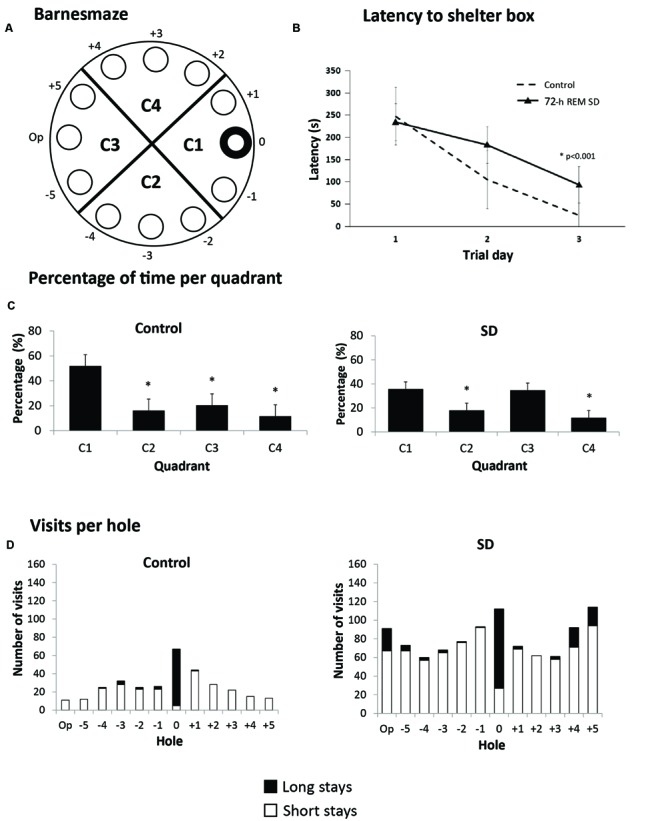
**Barnes maze. (A)** Schematic representation of the Barnes maze used; hole 0 indicates shelter hole. **(B)** Mean latency (5 assays per day) for 3 days. **(C)** Percentage of time per quadrant in the 72-h REM SD group vs. controls. Bars indicate mean ± SE. (^∗^) Indicates a significant difference (*p* < 0.05) (Mann–Whitney “*U*” test). **(D)** Number of visits to each hole in the last five assays. Upper part of each bar (black) represents the long visits (5 s or more), and lower part (white) are short visits (less than 5 s). Different strategies can be seen in each group according to the number of visits.

Hippocampus-dependent learning is affected immediately after a SD period (Yang et al., 2008; Hagewoud et al., 2010). However, it was unknown whether SD could produce a persistent cognitive impairment. For this purpose, we exposed mice to 72-h SD followed by biotery standard conditions for 21 days (*n* = 6 per group). After this period, we evaluated spatial memory in the Barnes maze (**Figure 3A**). The purpose was to evaluate the capacity of solving a spatial memory task after a long-term recovery of REM SD. The 72-h REM SD group showed statistically significant longer escaping latencies (94.0 ± 10.2 s) as compared to the control group (25.2 ± 4.5 s; *p* < 0.001, Mann–Whitney “*U*” test) (**Figure 3B**). These data suggest that both groups learned and completed the task, but the SD group showed a slower learning performance than controls. Since longer latencies in the target quadrant reveals better spatial learning (Conrad, 2010), we calculated the percentage of time spent on each quadrant. Our data indicated that the control group showed significantly more time in target quadrant (C1) than in the other quadrants. In contrast, the 72-h REM SD mice spent similar time in the C1 quadrant and the opposite quadrant (C3) as compared to controls, which supports the notion that SD impairs spatial memory (**Figure 3C**).

To analyze the strategy used by mice to solve this paradigm, we analyzed the frequency of visits to each hole. Visits were categorized as short visits (less than 5 s per hole) and long visits (5 or more seconds per hole) (**Figure 3D**). The control group had more visits to the shelter hole and a few number of visits to distant holes. In contrast, the 72-h REM SD group had approximately the same number of visits between the shelter and distant holes. We found that the control mice presented a high number of visits to the target hole as compared with the rest of the holes (*p* = 0.045, *U* = 5.5). In contrast, the SD animals did not show significant differences in the number of visits to the target hole when compared to the rest of the holes (*p* = 0.106, *U* = 8.0). This evidence indicated that SD animals solved the paradigm by exploring the maze serially, while the controls used a spatial strategy (**Figure 3D**). Taken together, these findings suggest that the 72-h REM SD group developed a long-term learning impairment.

### REM SD Impairs the Cellular Composition of the DG

**FIGURE 4:**
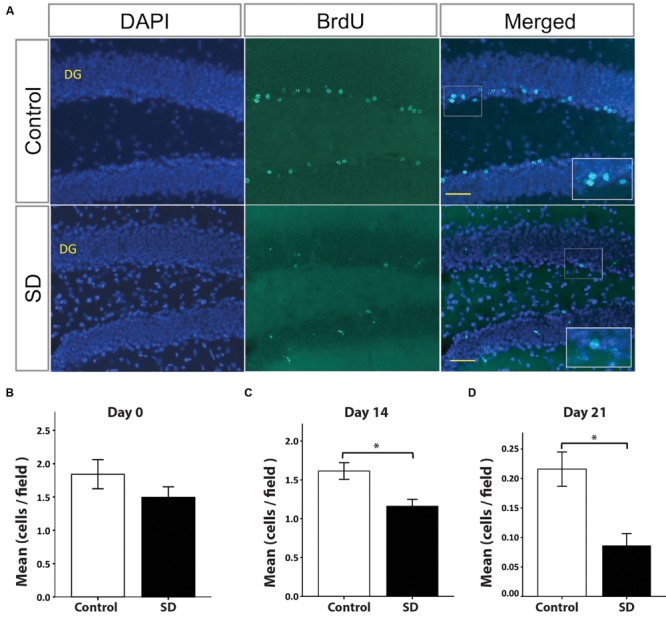
**BrdU+ cells in the dentate gyrus decrease in REM SD groups.** Representative pictures by immunofluorescence of the dentate gyrus (DG) of controls and the SD group **(A)**. The number of BrdU+ cells in DG (green) was significantly reduced by the effect of SD at different time points: day 0 **(B)**, day 14 **(C)**, and day 21 **(D)**. Bars indicate the mean of BrdU+ cells per field (445 μm^2^) ± SE. Asterisks Indicate statistical differences (Mann–Whitney “*U*” test). Nuclei are in blue by DAPI staining. Scale bar = 10 μm.

Spatial memory is associated with hippocampal neurogenesis (Kee et al., 2007). Sleep fragmentation and SD impair hippocampal-dependent memory and hippocampal neurogenesis (Guzman-Marin et al., 2003; Silva et al., 2004; Guzman-Marin et al., 2008; Mueller et al., 2008; Sportiche et al., 2010). However, it was unknown whether the cell fate of SGZ progenitors is affected over a long period of time after exposure to SD. For this purpose, we injected BrdU in the last day of REM SD and sacrificed animals (*n* = 5 per group) at different time points (**Figure 1**). At day 0 (BrdU injection 2-h before sacrifice), the number of BrdU+ cells was not statistically different between the REM SD group vs. the control group (1.8 ± 0.2 cells/field and 1.5 ± 0.2 cells /field, respectively). Remarkably, at day 14, we found a significant decrease in SD group (1.2 ± 0.1 cells /field) compared to the control (1.6 ± 0.1 cells /field; *p* = 0.001, Mann–Whitney “*U*” test). At day 21, BrdU+ cells also showed a significant decrease in the SD group (0.1 ± 0.02 cells/field) compared to the control group (0.2 ± 0.03 cells/field; *p* < 0.001) (**Figure 4**).

BrdU is a proliferation marker that indicates DNA synthesis (Gratzner, 1982), but it does not allow to identify the cellular phenotype. Nestin is a type IV intermediate filament expressed in the neural precursor cells of the SGZ (Michalczyk and Ziman, 2005). For this reason, we also assessed the number of double-labeled BrdU+/Nestin+ cells in the DG at day 0. Our results showed no significant differences between the REM SD group and controls (data not shown). To confirm whether BrdU-expressing cells found at day 14 corresponded to neuroblasts, we did a double immunohistochemistry for BrdU and DCX (a neuroblast marker). Our results showed a significant decrease in the number of BrdU+/DCX+ cells of the REM SD group (0.9 ± 0.1 cells/field, *p* = 0.003) when compared to the control group (control 1.4 ± 0.1 cells/field). To determine the number of mature granular neurons (NeuN+ cells) derived from the BrdU-labeled neural progenitors, we sacrificed animals 21 days after the REM SD. We found that the number of BrdU+NeuN+ in the SGZ did not show statistically significant differences between groups (controls = 0.16 ± 0.04 vs. REM SD = 0.1 ± 0.03 cells/field, *p* = 0.212, Mann–Whitney “*U*” test). Taken together, these data suggest that REM SD may affect the fate of cells generated in the adult SGZ.

### 72-h SD Can Produce Long-Term Apoptosis

**FIGURE 5:**
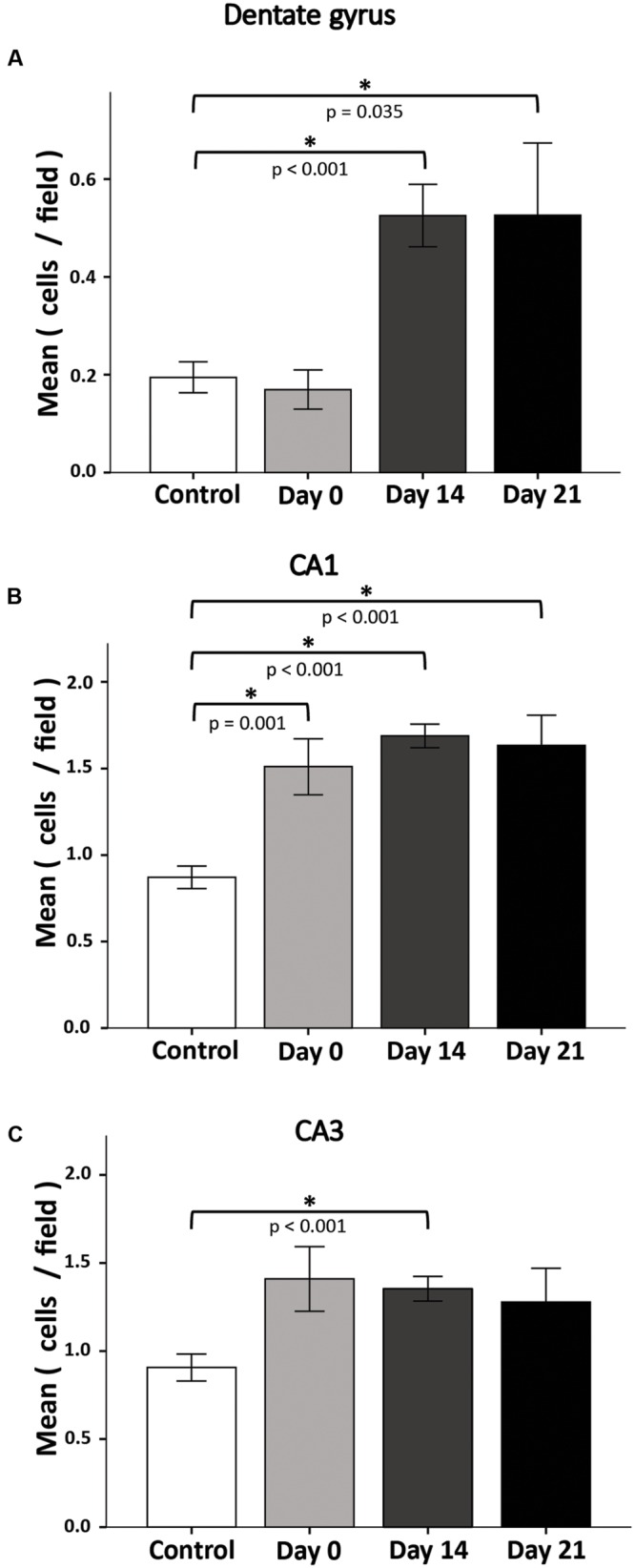
**Apoptosis rate in the DG(A), CA1(B), and CA3(C) after 72-h REM SD.** Plots represent the mean ± SE of TUNEL+ cells by field (445 μm^2^). Asterisks indicate significant differences (Mann–Whitney “*U*” test).

In response to stressful conditions neural cells trigger apoptotic signaling; SD may be considered a potentially stressful condition (Koban et al., 2006). To determine the long-term effects of 72-h REM SD, we analyzed the number of apoptotic cells (TUNEL+ cells) in the DG at different time points after SD (**Figure 5A**). At day 0, the number of apoptotic cells was similar between both groups: the control group (0.19 ± 0.03 cells /field) and the 72-h SD group (0.17 ± 0.04 cells /field). On day 14, the apoptosis rate increased significantly in the SD mice (0.53 ± 0.06 cells /field) as compared to controls (*p* < 0.001, Mann–Whitney “*U” test*). At day 21, apoptosis rate remained elevated in the SD animals (0.53 ± 0.15 cells/field) as compared to controls (*p* = 0.035, Mann–Whitney “*U*” test).

### Apoptosis in CA1 and CA3 Hippocampal Regions May Also Be Responsible for Spatial Memory Impairment

Interestingly, when assessing apoptosis in the DG we found notorious apoptotic labeling in other hippocampal regions. It is well known that different hippocampal structures are responsible for different functions, i.e., DG is responsible for pattern separation (Kesner, 2007; Treves et al., 2008) and CA1 for LTP (McHugh et al., 1996). In order to know whether REM SD is affecting other hippocampal regions, we decided to assess apoptosis in CA1 and CA3 hippocampal regions (**Figures 5B,C**). The number of apoptotic cells in CA1 region showed a significant increase in all groups when compared to controls. On day 0 in the SD group apoptosis increased significantly (control 0.87 ± 0.07, SD 1.5 ± 0.16, *p* = 0.001). On day 14, apoptosis rate in SD animals (SD 1.7 ± 0.07) was significantly higher than the control group (*p* < 0.001). A similar event was observed on day 21 (SD mice 1.6 ± 0.17; *p* < 0.001, Mann–Whitney “*U*” test). In the CA3 region, apoptosis labeling showed a significant difference at the day 14 only (control 0.9 ± 0.08, SD 1.4 ± 0.07, *p* < 0.001, Mann–Whitney “*U*” test) (**Figure 5C**). These findings could suggest that REM SD affects CA regions by apoptotic processes which could be related to spatial memory impairment.

### Insulin-Like Growth Factor 1 Receptor (IGF-1R)

**FIGURE 6:**
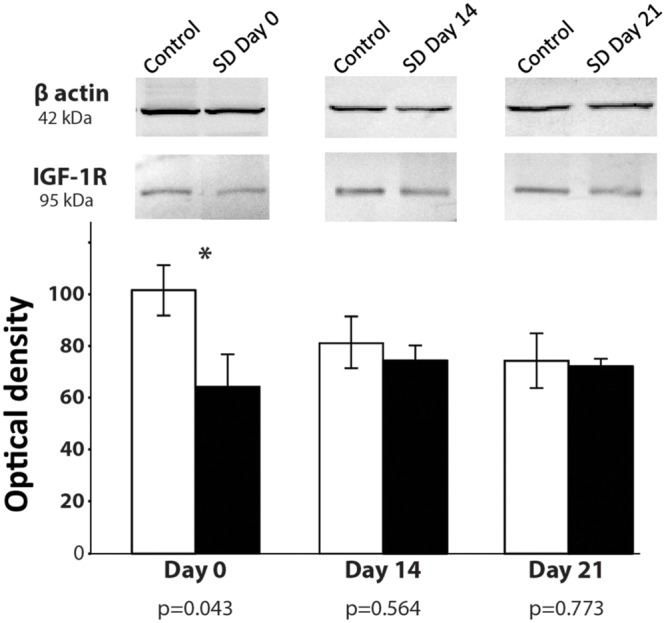
**p-IGF-1R expression after 74-h REM SD.** Graphics show the mean ± SE of the optical density for p-IGF-1R expression. Asterisks show significant difference. *SD* = 72-h REM sleep deprivation.

Stress induces significant changes in the cellular microenvironment by decreasing several essential factors, such as growth factors (Lucassen et al., 2010). IGF-1 stimulates the proliferation of neural precursors and increases survival of neurons and oligodendrocytes (D’Ercole et al., 2002). Phosphorylation of IGF-1R triggers cell growth and survival in neural cells. To determine whether SD generates changes in phosphorylated IGF-1 receptors (p-IGF-1R), we sacrificed animals at day 0, 14, and 21 days after 72-h REM SD and analyzed the p-IGF-1R expression by Western Blot in the hippocampus. On day 0, we found a significant decrease in the expression of p-IGF-1R in the 72-h REM SD animals (64.6 ± 12.2) as compared to the control group (102.0 ± 9.8, *p* = 0.043, Mann–Whitney “*U*” test) (**Figure 6**). At day 14, we did not find statistically significant differences between the control group (80.9 ± 9.9) vs. the 72-h REM SD group (74.1 ± 5.5). Similar findings were obtained at day 21 (controls = 73.9 ± 10.5 vs. SD mice = 71.7 ± 2.9) (**Figure 6**). To establish the existence of significant differences in IGFR1 expression in the control group, we did a statistical analysis among controls. We did not find statistically significant differences in the IGFR1 expression among controls (Day 0 vs. Day 14, *p* = 0.248, *U* = 4.0; Day 0 vs. Day 21, *p* = 0.083, *U* = 2.0). This evidence indicates that the statistical differences between controls and the SD group observed at day 0 were due to a reduction in the expression of IGFR1 in the SD animals, instead of an increase in the control expression. Nevertheless, we cannot fully discard that a slightly increase in the control group is also occurring. These data indicates that the 72-h SD decreases transiently the expression of p-IGF-1R, which returns to the basal levels after sleep recovery.

## Discussion

To the best of our knowledge, previous studies that analyzed the effects of SD had been performed during or immediately after the SD period, but none of them had reported the long-term implications of SD. In this study, we showed that 72-h of REM SD produced long-lasting spatial memory impairment, which could be associated with the reduction of BrdU+ cells found in the SGZ and the elevated hippocampal apoptosis rate in the SD animals after a period of recovery sleep. Taken together, our findings indicate that SD for 72-h can induce deleterious effects on behavior and the SGZ that persist for at least 3 weeks.

### 72-h of REM SD Impairs Spatial Memory for a Long Period of Time

Most studies in rats show deterioration in cognitive performance, such as memory encoding and consolidation, immediately after SD exposure (Ward et al., 2009; Hagewoud et al., 2010). To date it was unknown whether a relatively short (72 h) REM SD affected the hippocampus-dependent memory over a long period of time. The Barnes Maze is based on the principle that rodents prefer closed dark places, so the animal has to learn to find the shelter guided by spatial cues. In our study, we found that the group exposed to 72-h of REM SD showed a significant delay in finding the shelter hole in the Barnes maze and used a non-spatial strategy to solve the maze. This behavior indicates a deficit in hippocampal-dependent learning and the use of non-hippocampal dependent strategies to find the target (Conrad, 2010). Thus, our findings indicate that cognitive disturbance produced by REM SD can remain for at least 21 days. The action mechanism involved in this memory impairment remains unclear.

### CORT Levels Fluctuate during REM SD

Sleep deprivation is considered a stressful situation (Koban et al., 2006; Zammit, 2007). Stress activates the HPA and produces an anxiety behavior (Schoenfeld and Gould, 2012). Glucocorticoids could affect neurogenesis by altering cell proliferation, differentiation and maturation in the SGZ (Wong and Herbert, 2006). In the present study, we quantified the serum CORT levels, one of the main stress biomarkers. Although, the statistical analysis did not show significant differences at different exposure times, we found an increase tendency at 24 and 48 h of REM SD as compared to controls. Interestingly, at 72-h of REM SD, the serum CORT levels were similar to the control group. These data are in accordance with previous reports that found no changes or even decrease in the CORT levels after 48 h of vigilance or longer (Kant et al., 1984; Guzman-Marin et al., 2008). Some studies have reported increase in CORT levels during REM SD night and the following day of prolonged awakening, which reflects an effort of maintaining awake state (Leproult et al., 1997; Chapotot et al., 2001). These events can be explained by a biphasic response of HPA axis that it would be activated against SD stress, while the prolonged wakefulness sleep would cause “weakening” on the activity of HPA axis (Chapotot et al., 2001).

### DG Cellular Composition Is Altered over a Long Period of Time by 72-h REM SD

The optimal functioning of DG requires continuous production of new neurons and their incorporation into the hippocampal circuitry (Kempermann et al., 2015). In our study, we injected a single dose of BrdU (100 mg /kg) 2-h before ending SD. We chose this experimental approach because it labels a small number of dividing cells in the SGZ, which allows doing a reliable quantification of BrdU+ cells in the brain tissue. However, this method has a potential pitfall: the continuous apoptosis in the SGZ conveys a very important reduction in the number of BrdU+ cells in this region (Dayer et al., 2003). Therefore, a large proportion of new neurons may die before reaching maturity (Gould et al., 1990; Biebl et al., 2000; Sierra et al., 2010). Thus, we believe that this phenomenon may explain the less number of BrdU+ cells observed in both groups at day 21 of the study. Our data suggest that the cell fate are impaired after sleep fragmentation or SD, which reinforces the notion that sleep stimulates the production of new neurons in the SGZ (Mirescu et al., 2006; Sportiche et al., 2010). However, the mechanism that causes this alteration in the SGZ precursor cells cannot be elucidated by our experimental approach.

Prolonged sleep fragmentation reduces the number of cells that express neuronal phenotype (Guzman-Marin et al., 2005, 2007, 2008). Hence, SD could affect the ability of SGZ progenitors to differentiate into mature neurons as observed in learning tasks (Hairston et al., 2005). We found that a single exposition to 72-h REM SD produces a long-lasting decrease in the number of BrdU+ cells in the SGZ observed 14 and 21 days after SD. Taken together; this evidence suggests that differentiation and maturation processes are more susceptible to sleep loss than proliferation. Our findings also indicated that SD induced transient changes in IGF-1R expression. Therefore, the long-lasting changes observed in cell fate and differentiation could not be attributed to IGF-1R modifications, but further studies are required to address this question.

Sleep deprivation–induced apoptosis may involve excitotoxicity by supraphysiological stimulation of ionotropic glutamate receptors (AMPAr and NMDAr). To our knowledge, apoptosis had not been described in hippocampus after REM SD, however, McDermott et al. (2003) and Chen et al. (2006) through their electrophysiological studies found that after 24–72-h REM SD there is a lower NMDAr:AMPAr synapse rate in CA1 and DG in hippocampus. Apoptosis is also involved in excitotoxicity that implies supraphysiological stimulation of several glutamate receptors, especially ionotropic (NMDA, AMPA, and KA). The activation of these receptors could lead to a massive cell depolarization resulting in a cytosolic Ca^2+^ overload that triggers cell death (Gasque, 2010), therefore a decrease in hippocampal connections.

### Apoptosis and REM SD

Apoptosis and some other cellular responses can be induced through the activation of GR to start genomic and non-genomic effects (Lu et al., 2007), and induce apoptosis via the intrinsic apoptotic pathway (Smith and Cidlowski, 2010). Persistent glucocorticoid activation may decrease hippocampal cell proliferation and trigger apoptosis by promoting the expression of pro-apoptotic gene *Bax* and suppression of *Bcl-2*. In our study, we did not find a significant increase of apoptotic cells in the DG immediately after SD. However, after 14 or 21-day recovery period, the number of apoptotic cell significantly increased when compared to the control group. Persistent apoptosis has been associated with long-lasting expression of the pro-apoptotic protein BAX in the prefrontal cortex of SD animals after a 24-h recovery period (Montes-Rodriguez et al., 2009).

### IGF-1R and REM SD

IGF-1 stimulates adult neural stem cells and drives neuronal differentiation (Aberg et al., 2003). In mammals, glucocorticoids reduce IGF-1 blood levels and IGF-1 gene expression by attenuating the synthesis of GH receptor (McCarthy et al., 1990; Unterman et al., 1993; Delany et al., 2001). Glucocorticoids also increase IGFBP-1 and IGFBP-2, whereas decrease IGFBP-3 that, in consequence, may inhibit IGF-1 function (Unterman et al., 1993; Okazaki et al., 1994; Rodgers et al., 1995). In mammals, IGFBP-1 production is up-regulated by glucocorticoids and down-regulated by insulin, which acts as a growth inhibitor by preventing IGFs from reaching their target receptors (Unterman et al., 1993; Rodgers et al., 1995). Therefore, the rapid increases in these IGFBPs induced by cortisol presumably inhibit IGF-1 action, which contributes to the cortisol-induced growth retardation. In the present study, we found a reduction in the expression of phosphorylated-IGF-1R in the animals that were sacrificed immediately after SD. These changes could be attributed to fluctuation in glucocorticoid levels that has been reported in SD animals (Galvez-Contreras et al., 2016). IGF-1 has a rapid mechanism of action and a short biological half-life (Rabinovsky et al., 2003). Thus, we speculate that this may explain the absence of changes in the IGF-1R levels found at days 14 and 21.

In summary, the classical point of view suggested that sleep recovery for a few hours could also recover the brain homeostasis. However, present evidence indicates that REM SD for 72-h can impair spatial memory, promote apoptosis and alter the fate of cells in the SGZ over a long period of time. Yet, further studies are needed to elucidate the mechanisms that can trigger these events.

## Author Contributions

All authors had full access to all the data in the study and take responsibility for the integrity of the data and the accuracy of the data analysis. Acquisition and interpretation of data, statistical analysis and, first draft of the manuscript: SS-R and RG-C Administrative, technical, and material support: SL, RR-Z, FJ-H, and GL-A. Study design, funding and supervision of the study: OG-P and RG-C.

## Conflict of Interest Statement

The authors declare that the research was conducted in the absence of any commercial or financial relationships that could be construed as a potential conflict of interest.

The reviewer RG and handling Editor declared their shared national institution, and the handling Editor states that the process nevertheless met the standards of a fair and objective review.

## Abbreviations

BDNF: brain-derived neurotrophic factor
BrdU: bromodeoxyuridine
CA1: cornus ammonis 1
CA3: cornus ammonis 3
CORT: corticosterone
DCX: doublecortin
DG: dentate gyrus
GH: growth hormone
GR: glucocorticoid receptors
HPA: hypothalamic-pituitary-adrenal axis
IGF-1: insulin-like growth factor 1
IGF-1R: insulin-like growth factor receptor
IGFBPs: IGF-binding proteins
LTP: long term potentiation
NeuN: neuronal nuclei
NGF: nerve growth factor
REM: rapid eye movement
SD: sleep deprivation
SGZ: subgranular zone
TUNEL: terminal deoxynucleotidyl transferase dUTP nick end labeling
